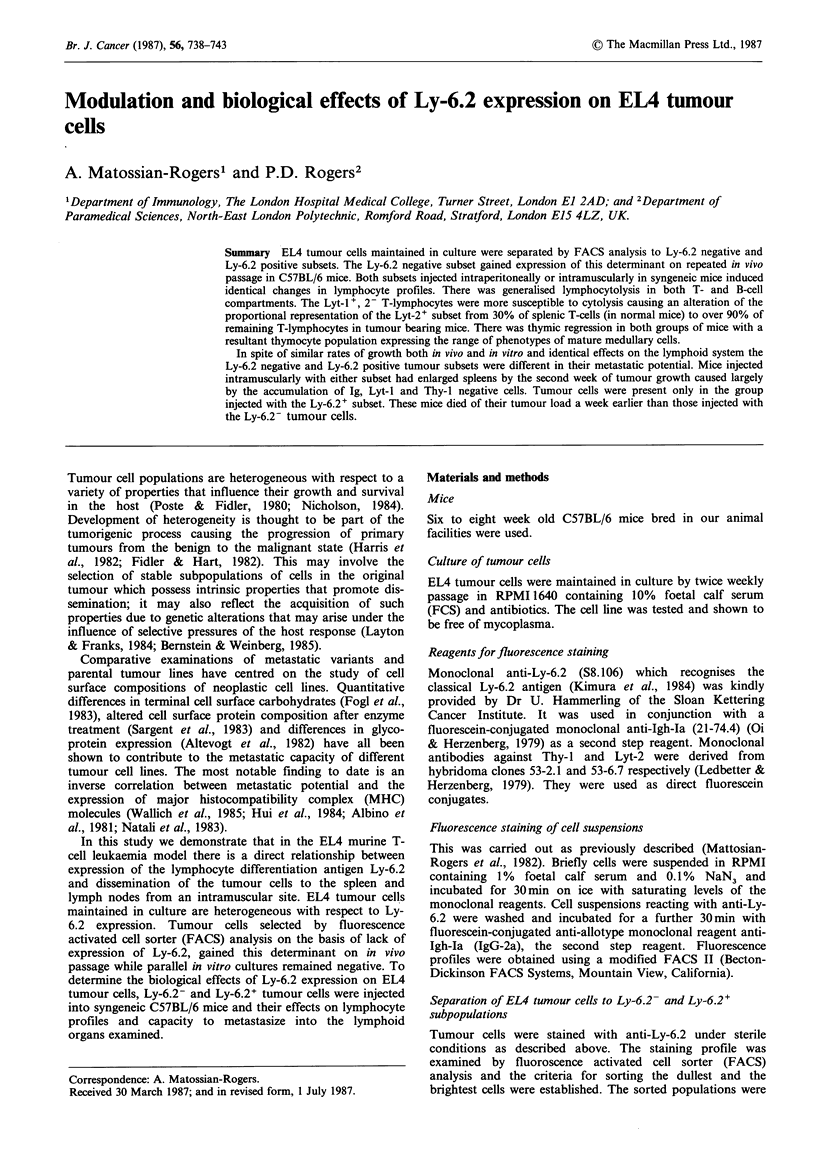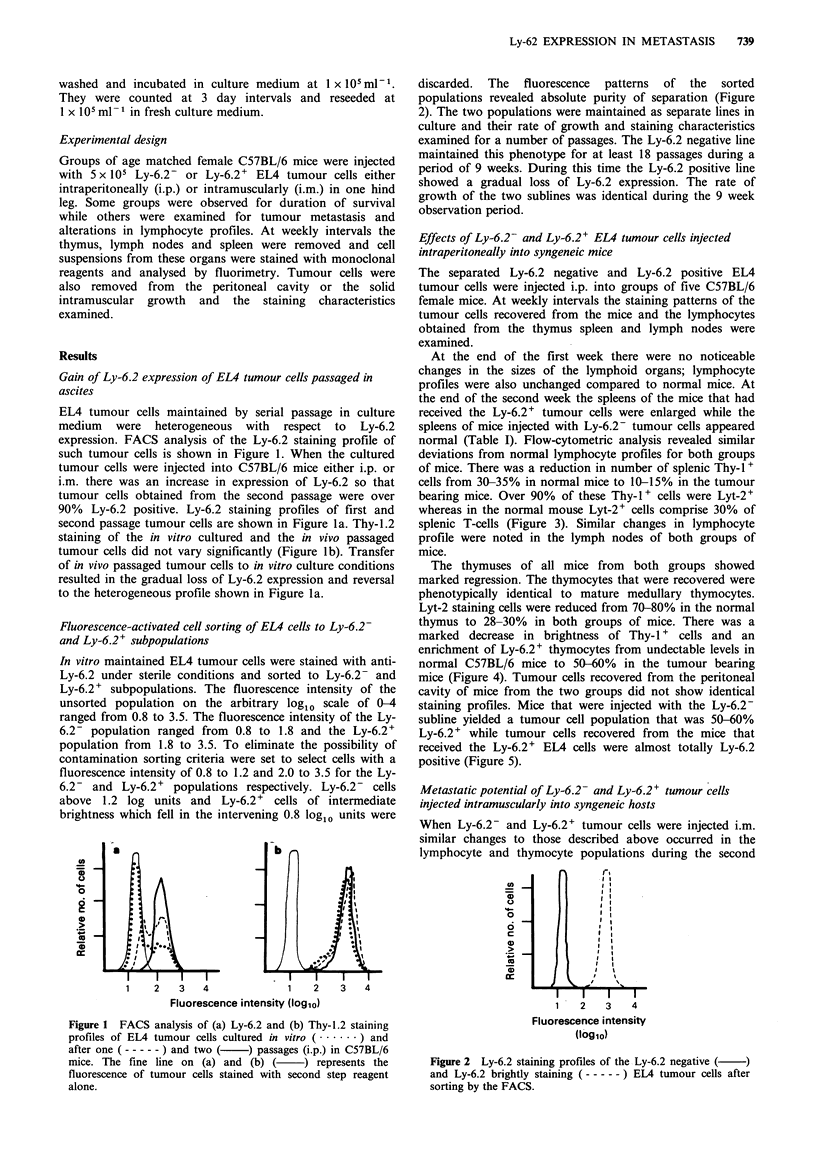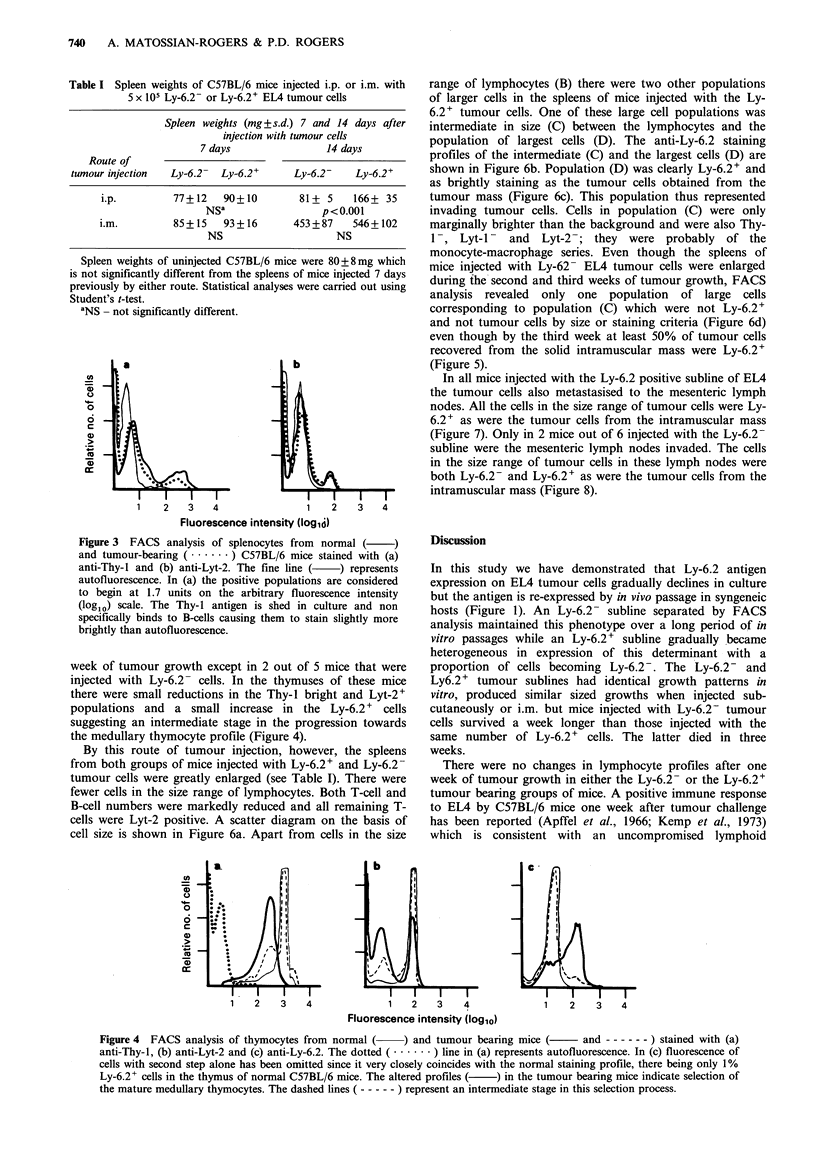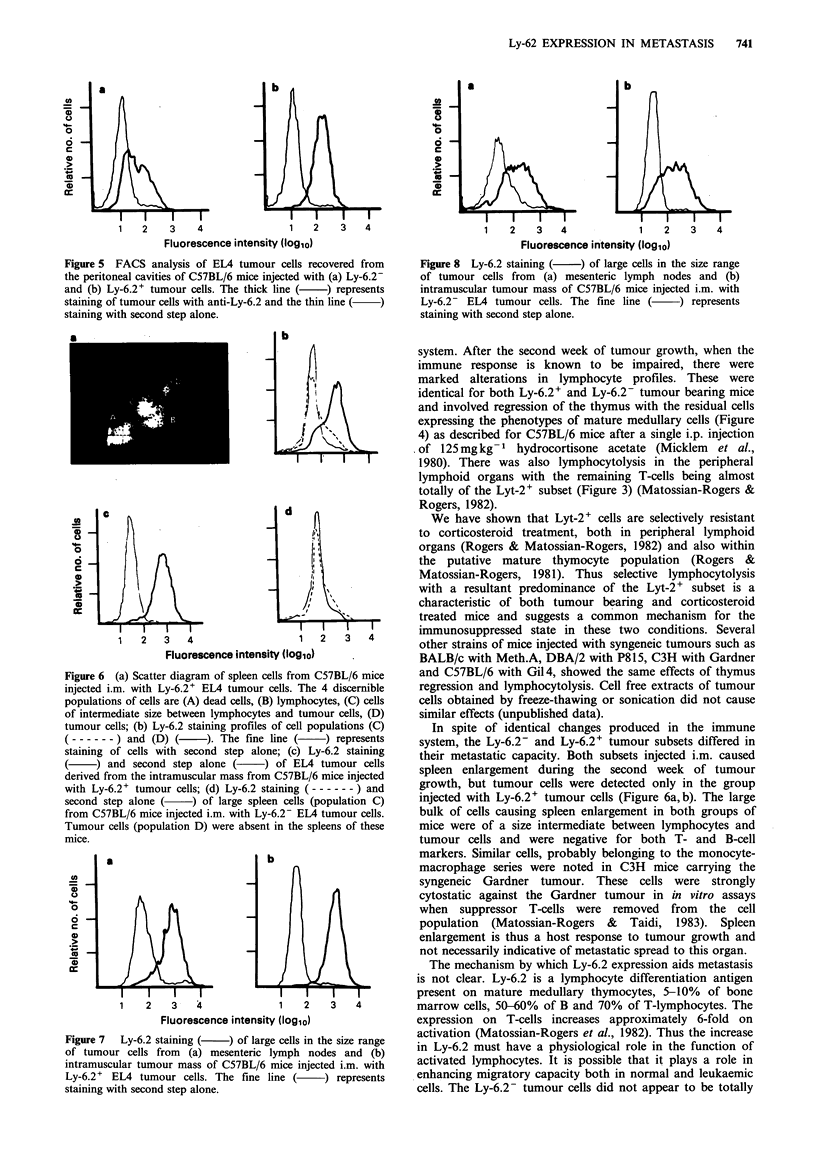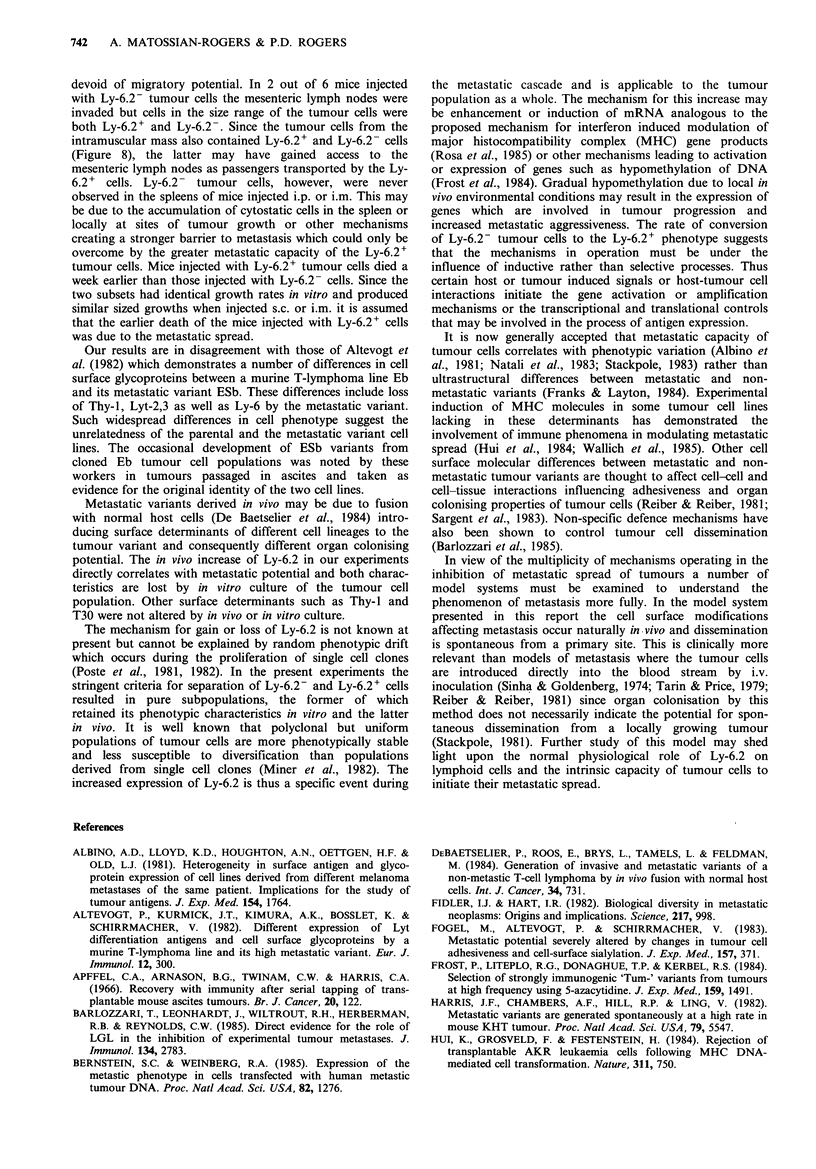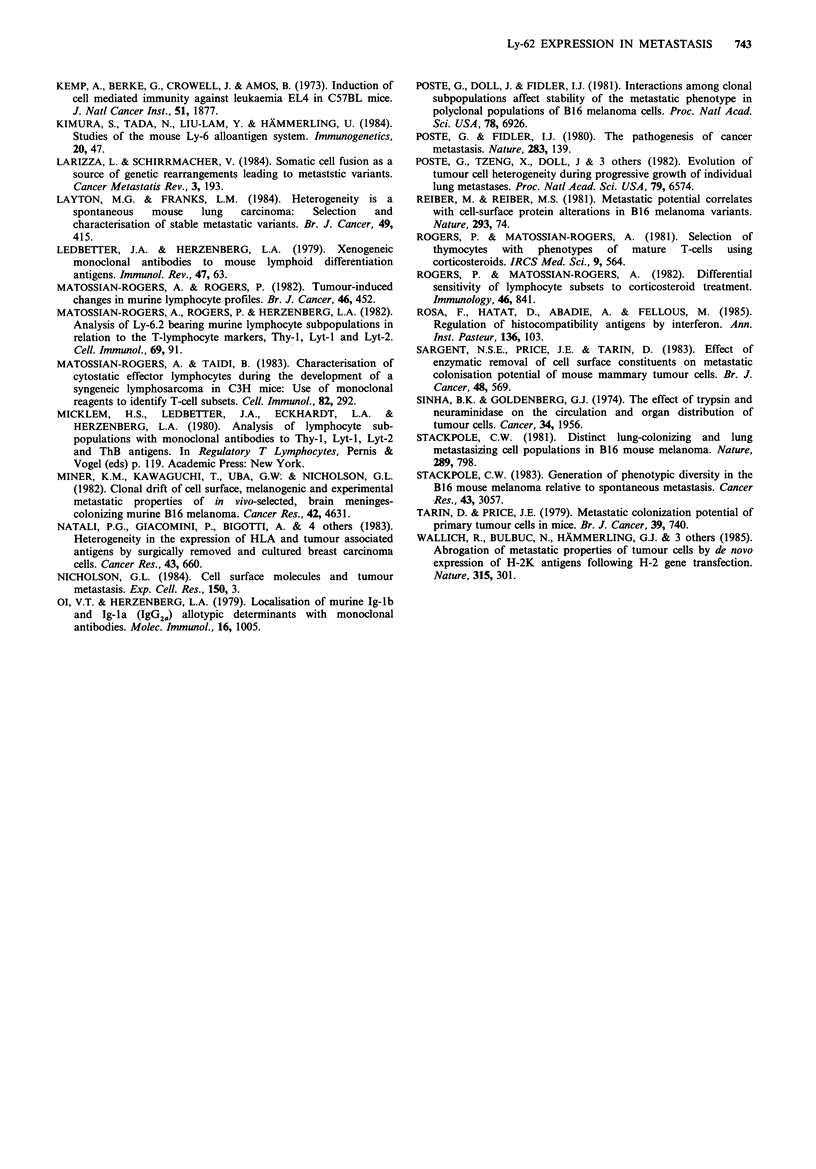# Modulation and biological effects of Ly-6.2 expression on EL4 tumour cells.

**DOI:** 10.1038/bjc.1987.280

**Published:** 1987-12

**Authors:** A. Matossian-Rogers, P. D. Rogers

**Affiliations:** Department of Immunology, London Hospital Medical College, UK.

## Abstract

EL4 tumour cells maintained in culture were separated by FACS analysis to Ly-6.2 negative and Ly-6.2 positive subsets. The Ly-6.2 negative subset gained expression of this determinant on repeated in vivo passage in C57BL/6 mice. Both subsets injected intraperitoneally or intramuscularly in syngeneic mice induced identical changes in lymphocyte profiles. There was generalised lymphocytolysis in both T- and B-cell compartments. The Lyt-1+, 2- T-lymphocytes were more susceptible to cytolysis causing an alteration of the proportional representation of the Lyt-2+ subset from 30% of splenic T-cells (in normal mice) to over 90% of remaining T-lymphocytes in tumour bearing mice. There was thymic regression in both groups of mice with a resultant thymocyte population expressing the range of phenotypes of mature medullary cells. In spite of similar rates of growth both in vivo and in vitro and identical effects on the lymphoid system the Ly-6.2 negative and Ly-6.2 positive tumour subsets were different in their metastatic potential. Mice injected intramuscularly with either subset had enlarged spleens by the second week of tumour growth caused largely by the accumulation of Ig, Lyt-1 and Thy-1 negative cells. Tumour cells were present only in the group injected with the Ly-6.2+ subset. These mice died of their tumour load a week earlier than those injected with the Ly-6.2- tumour cells.


*Br. J. Cancer* (1987), **56**, 738–743 © The Macmillan Press Ltd., 1987

### Modulation and biological effects of Ly-6.2 expression on EL4 tumour cells

A. Matossian-Rogers[1] and P.D. Rogers[2]

[1]*Department of Immunology, The London Hospital Medical College, Turner Street, London E1 2AD; and* [2]*Department of Paramedical Sciences, North-East London Polytechnic, Romford Road, Stratford, London E15 4LZ, UK.*

**Summary** EL4 tumour cells maintained in culture were separated by FACS analysis to Ly-6.2 negative and Ly-6.2 positive subsets. The Ly-6.2 negative subset gained expression of this determinant on repeated *in vivo* passage in C57BL/6 mice. Both subsets injected intraperitoneally or intramuscularly in syngeneic mice induced identical changes in lymphocyte profiles. There was generalised lymphocytolysis in both T- and B-cell compartments. The Lyt-1$^+$, 2$^-$ T-lymphocytes were more susceptible to cytolysis causing an alteration of the proportional representation of the Lyt-2$^+$ subset from 30% of splenic T-cells (in normal mice) to over 90% of remaining T-lymphocytes in tumour bearing mice. There was thymic regression in both groups of mice with a resultant thymocyte population expressing the range of phenotypes of mature medullary cells.

In spite of similar rates of growth both *in vivo* and *in vitro* and identical effects on the lymphoid system the Ly-6.2 negative and Ly-6.2 positive tumour subsets were different in their metastatic potential. Mice injected intramuscularly with either subset had enlarged spleens by the second week of tumour growth caused largely by the accumulation of Ig, Lyt-1 and Thy-1 negative cells. Tumour cells were present only in the group injected with the Ly-6.2$^+$ subset. These mice died of their tumour load a week earlier than those injected with the Ly-6.2$^-$ tumour cells.

Tumour cell populations are heterogeneous with respect to a variety of properties that influence their growth and survival in the host (Poste & Fidler, 1980; Nicholson, 1984). Development of heterogeneity is thought to be part of the tumorigenic process causing the progression of primary tumours from the benign to the malignant state (Harris *et al.*, 1982; Fidler & Hart, 1982). This may involve the selection of stable subpopulations of cells in the original tumour which possess intrinsic properties that promote dissemination; it may also reflect the acquisition of such properties due to genetic alterations that may arise under the influence of selective pressures of the host response (Layton & Franks, 1984; Bernstein & Weinberg, 1985).

Comparative examinations of metastatic variants and parental tumour lines have centred on the study of cell surface compositions of neoplastic cell lines. Quantitative differences in terminal cell surface carbohydrates (Fogl *et al.*, 1983), altered cell surface protein composition after enzyme treatment (Sargent *et al.*, 1983) and differences in glycoprotein expression (Altevogt *et al.*, 1982) have all been shown to contribute to the metastatic capacity of different tumour cell lines. The most notable finding to date is an inverse correlation between metastatic potential and the expression of major histocompatibility complex (MHC) molecules (Wallich *et al.*, 1985; Hui *et al.*, 1984; Albino *et al.*, 1981; Natali *et al.*, 1983).

In this study we demonstrate that in the EL4 murine T-cell leukaemia model there is a direct relationship between expression of the lymphocyte differentiation antigen Ly-6.2 and dissemination of the tumour cells to the spleen and lymph nodes from an intramuscular site. EL4 tumour cells maintained in culture are heterogeneous with respect to Ly-6.2 expression. Tumour cells selected by fluorescence activated cell sorter (FACS) analysis on the basis of lack of expression of Ly-6.2, gained this determinant on *in vivo* passage while parallel *in vitro* cultures remained negative. To determine the biological effects of Ly-6.2 expression on EL4 tumour cells, Ly-6.2$^-$ and Ly-6.2$^+$ tumour cells were injected into syngeneic C57BL/6 mice and their effects on lymphocyte profiles and capacity to metastasize into the lymphoid organs examined.

Correspondence: A. Matossian-Rogers.
Received 30 March 1987; and in revised form, 1 July 1987.

### Materials and methods

### *Mice*

Six to eight week old C57BL/6 mice bred in our animal facilities were used.

### *Culture of tumour cells*

EL4 tumour cells were maintained in culture by twice weekly passage in RPMI 1640 containing 10% foetal calf serum (FCS) and antibiotics. The cell line was tested and shown to be free of mycoplasma.

### *Reagents for fluorescence staining*

Monoclonal anti-Ly-6.2 (S8.106) which recognises the classical Ly-6.2 antigen (Kimura *et al.*, 1984) was kindly provided by Dr U. Hammerling of the Sloan Kettering Cancer Institute. It was used in conjunction with a fluorescein-conjugated monoclonal anti-Igh-Ia (21-74.4) (Oi & Herzenberg, 1979) as a second step reagent. Monoclonal antibodies against Thy-1 and Lyt-2 were derived from hybridoma clones 53-2.1 and 53-6.7 respectively (Ledbetter & Herzenberg, 1979). They were used as direct fluorescein conjugates.

### *Fluorescence staining of cell suspensions*

This was carried out as previously described (Mattosian-Rogers *et al.*, 1982). Briefly cells were suspended in RPMI containing 1% foetal calf serum and 0.1% $NaN_3$ and incubated for 30 min on ice with saturating levels of the monoclonal reagents. Cell suspensions reacting with anti-Ly-6.2 were washed and incubated for a further 30 min with fluorescein-conjugated anti-allotype monoclonal reagent anti-Igh-Ia (IgG-2a), the second step reagent. Fluorescence profiles were obtained using a modified FACS II (Becton-Dickinson FACS Systems, Mountain View, California).

### *Separation of EL4 tumour cells to Ly-6.2$^-$ and Ly-6.2$^+$ subpopulations*

Tumour cells were stained with anti-Ly-6.2 under sterile conditions as described above. The staining profile was examined by fluoroscence activated cell sorter (FACS) analysis and the criteria for sorting the dullest and the brightest cells were established. The sorted populations were washed and incubated in culture medium at $1 \times 10^5 ml^{-1}$. They were counted at 3 day intervals and reseeded at $1 \times 10^5 ml^{-1}$ in fresh culture medium.

*Experimental design*

Groups of age matched female C57BL/6 mice were injected with $5 \times 10^5$ Ly-6.2$^-$ or Ly-6.2$^+$ EL4 tumour cells either intraperitoneally (i.p.) or intramuscularly (i.m.) in one hind leg. Some groups were observed for duration of survival while others were examined for tumour metastasis and alterations in lymphocyte profiles. At weekly intervals the thymus, lymph nodes and spleen were removed and cell suspensions from these organs were stained with monoclonal reagents and analysed by fluorimetry. Tumour cells were also removed from the peritoneal cavity or the solid intramuscular growth and the staining characteristics examined.

### Results

*Gain of Ly-6.2 expression of EL4 tumour cells passaged in ascites*

EL4 tumour cells maintained by serial passage in culture medium were heterogeneous with respect to Ly-6.2 expression. FACS analysis of the Ly-6.2 staining profile of such tumour cells is shown in Figure 1. When the cultured tumour cells were injected into C57BL/6 mice either i.p. or i.m. there was an increase in expression of Ly-6.2 so that tumour cells obtained from the second passage were over 90% Ly-6.2 positive. Ly-6.2 staining profiles of first and second passage tumour cells are shown in Figure 1a. Thy-1.2 staining of the *in vitro* cultured and the *in vivo* passaged tumour cells did not vary significantly (Figure 1b). Transfer of *in vivo* passaged tumour cells to *in vitro* culture conditions resulted in the gradual loss of Ly-6.2 expression and reversal to the heterogeneous profile shown in Figure 1a.

*Fluorescence-activated cell sorting of EL4 cells to Ly-6.2$^-$ and Ly-6.2$^+$ subpopulations*

*In vitro* maintained EL4 tumour cells were stained with anti-Ly-6.2 under sterile conditions and sorted to Ly-6.2$^-$ and Ly-6.2$^+$ subpopulations. The fluorescence intensity of the unsorted population on the arbitrary $\log_{10}$ scale of 0–4 ranged from 0.8 to 3.5. The fluorescence intensity of the Ly-6.2$^-$ population ranged from 0.8 to 1.8 and the Ly-6.2$^+$ population from 1.8 to 3.5. To eliminate the possibility of contamination sorting criteria were set to select cells with a fluorescence intensity of 0.8 to 1.2 and 2.0 to 3.5 for the Ly-6.2$^-$ and Ly-6.2$^+$ populations respectively. Ly-6.2$^-$ cells above 1.2 log units and Ly-6.2$^+$ cells of intermediate brightness which fell in the intervening 0.8 $\log_{10}$ units were discarded. The fluorescence patterns of the sorted populations revealed absolute purity of separation (Figure 2). The two populations were maintained as separate lines in culture and their rate of growth and staining characteristics examined for a number of passages. The Ly-6.2 negative line maintained this phenotype for at least 18 passages during a period of 9 weeks. During this time the Ly-6.2 positive line showed a gradual loss of Ly-6.2 expression. The rate of growth of the two sublines was identical during the 9 week observation period.

*Effects of Ly-6.2$^-$ and Ly-6.2$^+$ EL4 tumour cells injected intraperitoneally into syngeneic mice*

The separated Ly-6.2 negative and Ly-6.2 positive EL4 tumour cells were injected i.p. into groups of five C57BL/6 female mice. At weekly intervals the staining patterns of the tumour cells recovered from the mice and the lymphocytes obtained from the thymus spleen and lymph nodes were examined.

At the end of the first week there were no noticeable changes in the sizes of the lymphoid organs; lymphocyte profiles were also unchanged compared to normal mice. At the end of the second week the spleens of the mice that had received the Ly-6.2$^+$ tumour cells were enlarged while the spleens of mice injected with Ly-6.2$^-$ tumour cells appeared normal (Table I). Flow-cytometric analysis revealed similar deviations from normal lymphocyte profiles for both groups of mice. There was a reduction in number of splenic Thy-1$^+$ cells from 30–35% in normal mice to 10–15% in the tumour bearing mice. Over 90% of these Thy-1$^+$ cells were Lyt-2$^+$ whereas in the normal mouse Lyt-2$^+$ cells comprise 30% of splenic T-cells (Figure 3). Similar changes in lymphocyte profile were noted in the lymph nodes of both groups of mice.

The thymuses of all mice from both groups showed marked regression. The thymocytes that were recovered were phenotypically identical to mature medullary thymocytes. Lyt-2 staining cells were reduced from 70–80% in the normal thymus to 28–30% in both groups of mice. There was a marked decrease in brightness of Thy-1$^+$ cells and an enrichment of Ly-6.2$^+$ thymocytes from undectable levels in normal C57BL/6 mice to 50–60% in the tumour bearing mice (Figure 4). Tumour cells recovered from the peritoneal cavity of mice from the two groups did not show identical staining profiles. Mice that were injected with the Ly-6.2$^-$ subline yielded a tumour cell population that was 50–60% Ly-6.2$^+$ while tumour cells recovered from the mice that received the Ly-6.2$^+$ EL4 cells were almost totally Ly-6.2 positive (Figure 5).

*Metastatic potential of Ly-6.2$^-$ and Ly-6.2$^+$ tumour cells injected intramuscularly into syngeneic hosts*

When Ly-6.2$^-$ and Ly-6.2$^+$ tumour cells were injected i.m. similar changes to those described above occurred in the lymphocyte and thymocyte populations during the second

**Figure 1** FACS analysis of (a) Ly-6.2 and (b) Thy-1.2 staining profiles of EL4 tumour cells cultured *in vitro* (· · · · · ·) and after one (- - - - -) and two (——) passages (i.p.) in C57BL/6 mice. The fine line on (a) and (b) (——) represents the fluorescence of tumour cells stained with second step reagent alone.

**Figure 2** Ly-6.2 staining profiles of the Ly-6.2 negative (——) and Ly-6.2 brightly staining (- - - - -) EL4 tumour cells after sorting by the FACS.

**Table I** Spleen weights of C57BL/6 mice injected i.p. or i.m. with $5 \times 10^5$ Ly-6.2⁻ or Ly-6.2⁺ EL4 tumour cells

| Route of tumour injection | *Spleen weights (mg±s.d.) 7 and 14 days after injection with tumour cells* 7 days Ly-6.2⁻ | 7 days Ly-6.2⁺ | 14 days Ly-6.2⁻ | 14 days Ly-6.2⁺ |
|---|---|---|---|---|
| i.p. | 77±12 | 90±10 | 81± 5 | 166± 35 |
| | NS[a] | | $p<0.001$ | |
| i.m. | 85±15 | 93±16 | 453±87 | 546±102 |
| | NS | | NS | |

Spleen weights of uninjected C57BL/6 mice were 80±8 mg which is not significantly different from the spleens of mice injected 7 days previously by either route. Statistical analyses were carried out using Student's *t*-test.
[a]NS – not significantly different.

**Figure 3** FACS analysis of splenocytes from normal (——) and tumour-bearing (······) C57BL/6 mice stained with (a) anti-Thy-1 and (b) anti-Lyt-2. The fine line (——) represents autofluorescence. In (a) the positive populations are considered to begin at 1.7 units on the arbitrary fluorescence intensity ($log_{10}$) scale. The Thy-1 antigen is shed in culture and non specifically binds to B-cells causing them to stain slightly more brightly than autofluorescence.

week of tumour growth except in 2 out of 5 mice that were injected with Ly-6.2⁻ cells. In the thymuses of these mice there were small reductions in the Thy-1 bright and Lyt-2⁺ populations and a small increase in the Ly-6.2⁺ cells suggesting an intermediate stage in the progression towards the medullary thymocyte profile (Figure 4).

By this route of tumour injection, however, the spleens from both groups of mice injected with Ly-6.2⁺ and Ly-6.2⁻ tumour cells were greatly enlarged (see Table I). There were fewer cells in the size range of lymphocytes. Both T-cell and B-cell numbers were markedly reduced and all remaining T-cells were Lyt-2 positive. A scatter diagram on the basis of cell size is shown in Figure 6a. Apart from cells in the size range of lymphocytes (B) there were two other populations of larger cells in the spleens of mice injected with the Ly-6.2⁺ tumour cells. One of these large cell populations was intermediate in size (C) between the lymphocytes and the population of largest cells (D). The anti-Ly-6.2 staining profiles of the intermediate (C) and the largest cells (D) are shown in Figure 6b. Population (D) was clearly Ly-6.2⁺ and as brightly staining as the tumour cells obtained from the tumour mass (Figure 6c). This population thus represented invading tumour cells. Cells in population (C) were only marginally brighter than the background and were also Thy-1⁻, Lyt-1⁻ and Lyt-2⁻; they were probably of the monocyte-macrophage series. Even though the spleens of mice injected with Ly-62⁻ EL4 tumour cells were enlarged during the second and third weeks of tumour growth, FACS analysis revealed only one population of large cells corresponding to population (C) which were not Ly-6.2⁺ and not tumour cells by size or staining criteria (Figure 6d) even though by the third week at least 50% of tumour cells recovered from the solid intramuscular mass were Ly-6.2⁺ (Figure 5).

In all mice injected with the Ly-6.2 positive subline of EL4 the tumour cells also metastasised to the mesenteric lymph nodes. All the cells in the size range of tumour cells were Ly-6.2⁺ as were the tumour cells from the intramuscular mass (Figure 7). Only in 2 mice out of 6 injected with the Ly-6.2⁻ subline were the mesenteric lymph nodes invaded. The cells in the size range of tumour cells in these lymph nodes were both Ly-6.2⁻ and Ly-6.2⁺ as were the tumour cells from the intramuscular mass (Figure 8).

### Discussion

In this study we have demonstrated that Ly-6.2 antigen expression on EL4 tumour cells gradually declines in culture but the antigen is re-expressed by *in vivo* passage in syngeneic hosts (Figure 1). An Ly-6.2⁻ subline separated by FACS analysis maintained this phenotype over a long period of *in vitro* passages while an Ly-6.2⁺ subline gradually became heterogeneous in expression of this determinant with a proportion of cells becoming Ly-6.2⁻. The Ly-6.2⁻ and Ly6.2⁺ tumour sublines had identical growth patterns *in vitro*, produced similar sized growths when injected sub-cutaneously or i.m. but mice injected with Ly-6.2⁻ tumour cells survived a week longer than those injected with the same number of Ly-6.2⁺ cells. The latter died in three weeks.

There were no changes in lymphocyte profiles after one week of tumour growth in either the Ly-6.2⁻ or the Ly-6.2⁺ tumour bearing groups of mice. A positive immune response to EL4 by C57BL/6 mice one week after tumour challenge has been reported (Apffel *et al.*, 1966; Kemp *et al.*, 1973) which is consistent with an uncompromised lymphoid

**Figure 4** FACS analysis of thymocytes from normal (——) and tumour bearing mice (—— and - - - - - -) stained with (a) anti-Thy-1, (b) anti-Lyt-2 and (c) anti-Ly-6.2. The dotted (······) line in (a) represents autofluorescence. In (c) fluorescence of cells with second step alone has been omitted since it very closely coincides with the normal staining profile, there being only 1% Ly-6.2⁺ cells in the thymus of normal C57BL/6 mice. The altered profiles (——) in the tumour bearing mice indicate selection of the mature medullary thymocytes. The dashed lines (- - - - -) represent an intermediate stage in this selection process.

**Figure 5** FACS analysis of EL4 tumour cells recovered from the peritoneal cavities of C57BL/6 mice injected with (a) Ly-6.2$^-$ and (b) Ly-6.2$^+$ tumour cells. The thick line (——) represents staining of tumour cells with anti-Ly-6.2 and the thin line (——) staining with second step alone.

**Figure 6** (a) Scatter diagram of spleen cells from C57BL/6 mice injected i.m. with Ly-6.2$^+$ EL4 tumour cells. The 4 discernible populations of cells are (A) dead cells, (B) lymphocytes, (C) cells of intermediate size between lymphocytes and tumour cells, (D) tumour cells; (b) Ly-6.2 staining profiles of cell populations (C) (- - - - - -) and (D) (——). The fine line (——) represents staining of cells with second step alone; (c) Ly-6.2 staining (——) and second step alone (——) of EL4 tumour cells derived from the intramuscular mass from C57BL/6 mice injected with Ly-6.2$^+$ tumour cells; (d) Ly-6.2 staining (- - - - - -) and second step alone (——) of large spleen cells (population C) from C57BL/6 mice injected i.m. with Ly-6.2$^-$ EL4 tumour cells. Tumour cells (population D) were absent in the spleens of these mice.

**Figure 7** Ly-6.2 staining (——) of large cells in the size range of tumour cells from (a) mesenteric lymph nodes and (b) intramuscular tumour mass of C57BL/6 mice injected i.m. with Ly-6.2$^+$ EL4 tumour cells. The fine line (——) represents staining with second step alone.

**Figure 8** Ly-6.2 staining (——) of large cells in the size range of tumour cells from (a) mesenteric lymph nodes and (b) intramuscular tumour mass of C57BL/6 mice injected i.m. with Ly-6.2$^-$ EL4 tumour cells. The fine line (——) represents staining with second step alone.

system. After the second week of tumour growth, when the immune response is known to be impaired, there were marked alterations in lymphocyte profiles. These were identical for both Ly-6.2$^+$ and Ly-6.2$^-$ tumour bearing mice and involved regression of the thymus with the residual cells expressing the phenotypes of mature medullary cells (Figure 4) as described for C57BL/6 mice after a single i.p. injection of 125 mg kg$^{-1}$ hydrocortisone acetate (Micklem *et al.*, 1980). There was also lymphocytolysis in the peripheral lymphoid organs with the remaining T-cells being almost totally of the Lyt-2$^+$ subset (Figure 3) (Matossian-Rogers & Rogers, 1982).

We have shown that Lyt-2$^+$ cells are selectively resistant to corticosteroid treatment, both in peripheral lymphoid organs (Rogers & Matossian-Rogers, 1982) and also within the putative mature thymocyte population (Rogers & Matossian-Rogers, 1981). Thus selective lymphocytolysis with a resultant predominance of the Lyt-2$^+$ subset is a characteristic of both tumour bearing and corticosteroid treated mice and suggests a common mechanism for the immunosuppressed state in these two conditions. Several other strains of mice injected with syngeneic tumours such as BALB/c with Meth.A, DBA/2 with P815, C3H with Gardner and C57BL/6 with Gil 4, showed the same effects of thymus regression and lymphocytolysis. Cell free extracts of tumour cells obtained by freeze-thawing or sonication did not cause similar effects (unpublished data).

In spite of identical changes produced in the immune system, the Ly-6.2$^-$ and Ly-6.2$^+$ tumour subsets differed in their metastatic capacity. Both subsets injected i.m. caused spleen enlargement during the second week of tumour growth, but tumour cells were detected only in the group injected with Ly-6.2$^+$ tumour cells (Figure 6a, b). The large bulk of cells causing spleen enlargement in both groups of mice were of a size intermediate between lymphocytes and tumour cells and were negative for both T- and B-cell markers. Similar cells, probably belonging to the monocyte-macrophage series were noted in C3H mice carrying the syngeneic Gardner tumour. These cells were strongly cytostatic against the Gardner tumour in *in vitro* assays when suppressor T-cells were removed from the cell population (Matossian-Rogers & Taidi, 1983). Spleen enlargement is thus a host response to tumour growth and not necessarily indicative of metastatic spread to this organ.

The mechanism by which Ly-6.2 expression aids metastasis is not clear. Ly-6.2 is a lymphocyte differentiation antigen present on mature medullary thymocytes, 5–10% of bone marrow cells, 50–60% of B and 70% of T-lymphocytes. The expression on T-cells increases approximately 6-fold on activation (Matossian-Rogers *et al.*, 1982). Thus the increase in Ly-6.2 must have a physiological role in the function of activated lymphocytes. It is possible that it plays a role in enhancing migratory capacity both in normal and leukaemic cells. The Ly-6.2$^-$ tumour cells did not appear to be totally devoid of migratory potential. In 2 out of 6 mice injected with Ly-6.2$^-$ tumour cells the mesenteric lymph nodes were invaded but cells in the size range of the tumour cells were both Ly-6.2$^+$ and Ly-6.2$^-$. Since the tumour cells from the intramuscular mass also contained Ly-6.2$^+$ and Ly-6.2$^-$ cells (Figure 8), the latter may have gained access to the mesenteric lymph nodes as passengers transported by the Ly-6.2$^+$ cells. Ly-6.2$^-$ tumour cells, however, were never observed in the spleens of mice injected i.p. or i.m. This may be due to the accumulation of cytostatic cells in the spleen or locally at sites of tumour growth or other mechanisms creating a stronger barrier to metastasis which could only be overcome by the greater metastatic capacity of the Ly-6.2$^+$ tumour cells. Mice injected with Ly-6.2$^+$ tumour cells died a week earlier than those injected with Ly-6.2$^-$ cells. Since the two subsets had identical growth rates *in vitro* and produced similar sized growths when injected s.c. or i.m. it is assumed that the earlier death of the mice injected with Ly-6.2$^+$ cells was due to the metastatic spread.

Our results are in disagreement with those of Altevogt *et al.* (1982) which demonstrates a number of differences in cell surface glycoproteins between a murine T-lymphoma line Eb and its metastatic variant ESb. These differences include loss of Thy-1, Lyt-2,3 as well as Ly-6 by the metastatic variant. Such widespread differences in cell phenotype suggest the unrelatedness of the parental and the metastatic variant cell lines. The occasional development of ESb variants from cloned Eb tumour cell populations was noted by these workers in tumours passaged in ascites and taken as evidence for the original identity of the two cell lines.

Metastatic variants derived *in vivo* may be due to fusion with normal host cells (De Baetselier *et al.*, 1984) introducing surface determinants of different cell lineages to the tumour variant and consequently different organ colonising potential. The *in vivo* increase of Ly-6.2 in our experiments directly correlates with metastatic potential and both characteristics are lost by *in vitro* culture of the tumour cell population. Other surface determinants such as Thy-1 and T30 were not altered by *in vivo* or *in vitro* culture.

The mechanism for gain or loss of Ly-6.2 is not known at present but cannot be explained by random phenotypic drift which occurs during the proliferation of single cell clones (Poste *et al.*, 1981, 1982). In the present experiments the stringent criteria for separation of Ly-6.2$^-$ and Ly-6.2$^+$ cells resulted in pure subpopulations, the former of which retained its phenotypic characteristics *in vitro* and the latter *in vivo*. It is well known that polyclonal but uniform populations of tumour cells are more phenotypically stable and less susceptible to diversification than populations derived from single cell clones (Miner *et al.*, 1982). The increased expression of Ly-6.2 is thus a specific event during the metastatic cascade and is applicable to the tumour population as a whole. The mechanism for this increase may be enhancement or induction of mRNA analogous to the proposed mechanism for interferon induced modulation of major histocompatibility complex (MHC) gene products (Rosa *et al.*, 1985) or other mechanisms leading to activation or expression of genes such as hypomethylation of DNA (Frost *et al.*, 1984). Gradual hypomethylation due to local *in vivo* environmental conditions may result in the expression of genes which are involved in tumour progression and increased metastatic aggressiveness. The rate of conversion of Ly-6.2$^-$ tumour cells to the Ly-6.2$^+$ phenotype suggests that the mechanisms in operation must be under the influence of inductive rather than selective processes. Thus certain host or tumour induced signals or host-tumour cell interactions initiate the gene activation or amplification mechanisms or the transcriptional and translational controls that may be involved in the process of antigen expression.

It is now generally accepted that metastatic capacity of tumour cells correlates with phenotypic variation (Albino *et al.*, 1981; Natali *et al.*, 1983; Stackpole, 1983) rather than ultrastructural differences between metastatic and non-metastatic variants (Franks & Layton, 1984). Experimental induction of MHC molecules in some tumour cell lines lacking in these determinants has demonstrated the involvement of immune phenomena in modulating metastatic spread (Hui *et al.*, 1984; Wallich *et al.*, 1985). Other cell surface molecular differences between metastatic and non-metastatic tumour variants are thought to affect cell–cell and cell–tissue interactions influencing adhesiveness and organ colonising properties of tumour cells (Reiber & Reiber, 1981; Sargent *et al.*, 1983). Non-specific defence mechanisms have also been shown to control tumour cell dissemination (Barlozzari *et al.*, 1985).

In view of the multiplicity of mechanisms operating in the inhibition of metastatic spread of tumours a number of model systems must be examined to understand the phenomenon of metastasis more fully. In the model system presented in this report the cell surface modifications affecting metastasis occur naturally *in vivo* and dissemination is spontaneous from a primary site. This is clinically more relevant than models of metastasis where the tumour cells are introduced directly into the blood stream by i.v. inoculation (Sinha & Goldenberg, 1974; Tarin & Price, 1979; Reiber & Reiber, 1981) since organ colonisation by this method does not necessarily indicate the potential for spontaneous dissemination from a locally growing tumour (Stackpole, 1981). Further study of this model may shed light upon the normal physiological role of Ly-6.2 on lymphoid cells and the intrinsic capacity of tumour cells to initiate their metastatic spread.

### References

ALBINO, A.D., LLOYD, K.D., HOUGHTON, A.N., OETTGEN, H.F. & OLD, L.J. (1981). Heterogeneity in surface antigen and glycoprotein expression of cell lines derived from different melanoma metastases of the same patient. Implications for the study of tumour antigens. *J. Exp. Med.* **154,** 1764.

ALTEVOGT, P., KURMICK, J.T., KIMURA, A.K., BOSSLET, K. & SCHIRRMACHER, V. (1982). Different expression of Lyt differentiation antigens and cell surface glycoproteins by a murine T-lymphoma line and its high metastatic variant. *Eur. J. Immunol.* **12,** 300.

APFFEL, C.A., ARNASON, B.G., TWINAM, C.W. & HARRIS, C.A. (1966). Recovery with immunity after serial tapping of transplantable mouse ascites tumours. *Br. J. Cancer,* **20,** 122.

BARLOZZARI, T., LEONHARDT, J., WILTROUT, R.H., HERBERMAN, R.B. & REYNOLDS, C.W. (1985). Direct evidence for the role of LGL in the inhibition of experimental tumour metastases. *J. Immunol.* **134,** 2783.

BERNSTEIN, S.C. & WEINBERG, R.A. (1985). Expression of the metastic phenotype in cells transfected with human metastic tumour DNA. *Proc. Natl Acad. Sci. USA,* **82,** 1276.

DEBAETSELIER, P., ROOS, E., BRYS, L., TAMELS, L. & FELDMAN, M. (1984). Generation of invasive and metastatic variants of a non-metastic T-cell lymphoma by *in vivo* fusion with normal host cells. *Int. J. Cancer,* **34,** 731.

FIDLER, I.J. & HART, I.R. (1982). Biological diversity in metastatic neoplasms: Origins and implications. *Science,* **217,** 998.

FOGEL, M., ALTEVOGT, P. & SCHIRRMACHER, V. (1983). Metastatic potential severely altered by changes in tumour cell adhesiveness and cell-surface sialylation. *J. Exp. Med.,* **157,** 371.

FROST, P., LITEPLO, R.G., DONAGHUE, T.P. & KERBEL, R.S. (1984). Selection of strongly immunogenic 'Tum-' variants from tumours at high frequency using 5-azacytidine. *J. Exp. Med.,* **159,** 1491.

HARRIS, J.F., CHAMBERS, A.F., HILL, R.P. & LING, V. (1982). Metastatic variants are generated spontaneously at a high rate in mouse KHT tumour. *Proc. Natl Acad. Sci. USA,* **79,** 5547.

HUI, K., GROSVELD, F. & FESTENSTEIN, H. (1984). Rejection of transplantable AKR leukaemia cells following MHC DNA-mediated cell transformation. *Nature,* **311,** 750.

KEMP, A., BERKE, G., CROWELL, J. & AMOS, B. (1973). Induction of cell mediated immunity against leukaemia EL4 in C57BL mice. *J. Natl Cancer Inst.*, **51**, 1877.

KIMURA, S., TADA, N., LIU-LAM, Y. & HÄMMERLING, U. (1984). Studies of the mouse Ly-6 alloantigen system. *Immunogenetics*, **20**, 47.

LARIZZA, L. & SCHIRRMACHER, V. (1984). Somatic cell fusion as a source of genetic rearrangements leading to metaststic variants. *Cancer Metastatis Rev.*, **3**, 193.

LAYTON, M.G. & FRANKS, L.M. (1984). Heterogeneity is a spontaneous mouse lung carcinoma: Selection and characterisation of stable metastatic variants. *Br. J. Cancer*, **49**, 415.

LEDBETTER, J.A. & HERZENBERG, L.A. (1979). Xenogeneic monoclonal antibodies to mouse lymphoid differentiation antigens. *Immunol. Rev.*, **47**, 63.

MATOSSIAN-ROGERS, A. & ROGERS, P. (1982). Tumour-induced changes in murine lymphocyte profiles. *Br. J. Cancer*, **46**, 452.

MATOSSIAN-ROGERS, A., ROGERS, P. & HERZENBERG, L.A. (1982). Analysis of Ly-6.2 bearing murine lymphocyte subpopulations in relation to the T-lymphocyte markers, Thy-1, Lyt-1 and Lyt-2. *Cell. Immunol.*, **69**, 91.

MATOSSIAN-ROGERS, A. & TAIDI, B. (1983). Characterisation of cytostatic effector lymphocytes during the development of a syngeneic lymphosarcoma in C3H mice: Use of monoclonal reagents to identify T-cell subsets. *Cell. Immunol.*, **82**, 292.

MICKLEM, H.S., LEDBETTER, J.A., ECKHARDT, L.A. & HERZENBERG, L.A. (1980). Analysis of lymphocyte subpopulations with monoclonal antibodies to Thy-1, Lyt-1, Lyt-2 and ThB antigens. In *Regulatory T Lymphocytes*, Pernis & Vogel (eds) p. 119. Academic Press: New York.

MINER, K.M., KAWAGUCHI, T., UBA, G.W: & NICHOLSON, G.L. (1982). Clonal drift of cell surface, melanogenic and experimental metastatic properties of *in vivo*-selected, brain meninges-colonizing murine B16 melanoma. *Cancer Res.*, **42**, 4631.

NATALI, P.G., GIACOMINI, P., BIGOTTI, A. & 4 others (1983). Heterogeneity in the expression of HLA and tumour associated antigens by surgically removed and cultured breast carcinoma cells. *Cancer Res.*, **43**, 660.

NICHOLSON, G.L. (1984). Cell surface molecules and tumour metastasis. *Exp. Cell. Res.*, **150**, 3.

OI, V.T. & HERZENBERG, L.A. (1979). Localisation of murine Ig-1b and Ig-1a ($IgG_{2a}$) allotypic determinants with monoclonal antibodies. *Molec. Immunol.*, **16**, 1005.

POSTE, G., DOLL, J. & FIDLER, I.J. (1981). Interactions among clonal subpopulations affect stability of the metastatic phenotype in polyclonal populations of B16 melanoma cells. *Proc. Natl Acad. Sci. USA*, **78**, 6926.

POSTE, G. & FIDLER, I.J. (1980). The pathogenesis of cancer metastasis. *Nature*, **283**, 139.

POSTE, G., TZENG, X., DOLL, J & 3 others (1982). Evolution of tumour cell heterogeneity during progressive growth of individual lung metastases. *Proc. Natl Acad. Sci. USA*, **79**, 6574.

REIBER, M. & REIBER, M.S. (1981). Metastatic potential correlates with cell-surface protein alterations in B16 melanoma variants. *Nature*, **293**, 74.

ROGERS, P. & MATOSSIAN-ROGERS, A. (1981). Selection of thymocytes with phenotypes of mature T-cells using corticosteroids. *IRCS Med. Sci.*, **9**, 564.

ROGERS, P. & MATOSSIAN-ROGERS, A. (1982). Differential sensitivity of lymphocyte subsets to corticosteroid treatment. *Immunology*, **46**, 841.

ROSA, F., HATAT, D., ABADIE, A. & FELLOUS, M. (1985). Regulation of histocompatibility antigens by interferon. *Ann. Inst. Pasteur*, **136**, 103.

SARGENT, N.S.E., PRICE, J.E. & TARIN, D. (1983). Effect of enzymatic removal of cell surface constituents on metastatic colonisation potential of mouse mammary tumour cells. *Br. J. Cancer*, **48**, 569.

SINHA, B.K. & GOLDENBERG, G.J. (1974). The effect of trypsin and neuraminidase on the circulation and organ distribution of tumour cells. *Cancer*, **34**, 1956.

STACKPOLE, C.W. (1981). Distinct lung-colonizing and lung metastasizing cell populations in B16 mouse melanoma. *Nature*, **289**, 798.

STACKPOLE, C.W. (1983). Generation of phenotypic diversity in the B16 mouse melanoma relative to spontaneous metastasis. *Cancer Res.*, **43**, 3057.

TARIN, D. & PRICE, J.E. (1979). Metastatic colonization potential of primary tumour cells in mice. *Br. J. Cancer*, **39**, 740.

WALLICH, R., BULBUC, N., HÄMMERLING, G.J. & 3 others (1985). Abrogation of metastatic properties of tumour cells by *de novo* expression of H-2K antigens following H-2 gene transfection. *Nature*, **315**, 301.